# Editorial: Promising therapeutic strategies for Alzheimer's disease: a focus on amyloid-β targeting

**DOI:** 10.3389/fnins.2024.1415641

**Published:** 2024-04-25

**Authors:** Alessandra Bigi, Ryan Limbocker, Cristina Cecchi

**Affiliations:** ^1^Department of Experimental and Clinical Biomedical Sciences, Section of Biochemistry, University of Florence, Florence, Italy; ^2^Department of Chemistry and Life Science, United States Military Academy, West Point, NY, United States

**Keywords:** neurodegeneration, amyloid aggregation, inflammation, tauroursodeoxycholic acid, neurotoxicity, memantine, NMDA—receptor

Alzheimer's disease (AD) is the most prevalent neurodegenerative disorder and the most common form of dementia, affecting about 55 million people worldwide (source: Alzheimer's Association). According to the amyloid hypothesis, the pathogenesis of AD is primarily triggered by the progressive accumulation and subsequent deposition of the amyloid-β (Aβ) peptide in the extracellular space of the brain cortex and hippocampus (Selkoe and Hardy, 2016). Specifically, the Aβ peptide is 40- or 42-residues long (Aβ_40_ and Aβ_42_, respectively) and arises after the pathogenic cleavage of the amyloid precursor protein (APP) by β- and γ-secretases (Selkoe and Hardy, 2016). Its abnormal aggregation further results in the formation of neurofibrillary tangles, composed of the hyperphosphorylated microtubule-associated Tau protein, culminating in neuronal and synaptic loss, finally leading to dementia (Selkoe and Hardy, 2016). The intracerebral accumulation of Aβ aggregates takes place at least two-three decades before the occurrence of clinical symptoms of AD (McDade et al., 2018), and this long delay renders the course of the disease progressively independent from Aβ deposition, thus explaining the poor clinical efficacy of the greatest majority of Aβ-targeting clinical trials (Golde et al., 2018). Such trials should be thus initiated before the development of symptoms, in a therapeutic window allocated in the preclinical phase, at which the blockage of Aβ aggregation should have its maximal benefit (Golde et al., 2018). This explains the primary interest of research in identifying early disease-modifying therapies aimed at targeting Aβ peptide in order to prevent its neurotoxicity.

The Research Topic entitled “*Promising therapeutic strategies for Alzheimer's disease: a focus on amyloid-*β *targeting*” published in Frontiers in Neuroscience includes four contributions: three original articles and one review providing new information on recently proposed and possible anti-Aβ therapies. In the first original research article, Noel et al. investigated the beneficial effect of focused ultrasound (FUS) paired with systemically-introduced microbubbles, a non-invasive technique for targeted and transient blood–brain barrier opening (BBBO), on anxiety, memory and AD-associated protein levels in triple transgenic (3xTg) AD mice treated at an early age and disease state. Importantly, this technique was previously demonstrated to reduce Aβ and tau accumulation, and to improve memory in models of late-stage AD (Noel et al., 2023). The study demonstrated that the repeated intervention with FUS-BBBO prevented the pathological accumulation of both Aβ and Tau, decreased anxiety and prevented cognitive decline in AD mice, thus pointing out this approach as a putative non-invasive technique to delay the onset and progression of pathology for carriers of AD-associated mutations. Similar approaches could hypothetically fill the lack of effective therapies for AD, or even prevent neurodegeneration with an early therapeutic intervention.

Memantine, a N-methyl-D-aspartate (NMDA) receptor antagonist is currently one of the few drugs approved by the Food and Drug Administration (FDA) and the European Medicines Agency (EMA) for the symptomatic treatment of moderate and severe AD (Tang et al., 2023). This molecule is the object of the research report by Rozumna et al., in which researchers shed light into its beneficial effect on hippocampal neurons. The clear potential of memantine as a neuroprotective agent against NMDA and Aβ-induced neurotoxicity was confirmed by the experimental work, reinforcing the previously reported significance of NMDA receptors and their associated excitotoxicity in AD (Cascella et al., 2017).

The pathogenesis of AD is extremely complex, involving a plethora of different pathways, cellular functions, organelles and cell types and, in this context, inflammation plays a pivotal role. This is why Kot et al. used peripheral blood mononuclear cells (PBMCs) to assess changes in pro-inflammatory cytokines released in response to recombinant Aβ_42_ and on the concentration of endogenous Aβ_40_. Researchers revealed a significant accumulation of Aβ_40_ in the cytoplasm, together with the aggregation of Aβ_42_ on the outer surface of the cell plasma membrane upon treatment, with β1 integrins playing a proamyloidogenic and proinflammation role.

In the final article of this Research Topic, Song et al. review the impact of bile acid metabolism, in particular the endogenous bile acid tauroursodeoxycholic acid, for its potential to reduce Aβ toxicity through several potential mechanisms, to include inhibiting Aβ deposition, regulating apoptotic pathways, preventing tau hyperphosphorylation, protecting neuronal synapses, or through its anti-inflammatory properties.

Collectively, this Research Topic represents an important update on promising current therapeutic approaches, with focus on specific mechanisms underlying the pathogenesis and progression of AD. Further progress is urgently needed to better understand the role of Aβ in AD, to include (1) enhanced diagnostics that can identify patients early in the course of AD, therein allowing their timely treatment prior to the presence of irreversible damage in their brains, (2) improved *in vitro* methods that form the types of intermediate and terminal amyloid species found in the human AD brain, therein recapitulating AD pathology, and (3) the development of cost-effective and more potent anti-amyloid therapeutics that build upon the recent momentum of the FDA-approved anti-amyloid-β monoclonal antibodies.

## Author contributions

AB: Writing – original draft, Writing – review & editing. RL: Writing – original draft, Writing – review & editing. CC: Writing – original draft, Writing – review & editing.
